# Integration of multiomics features for blood-based early detection of colorectal cancer

**DOI:** 10.1186/s12943-024-01959-3

**Published:** 2024-08-22

**Authors:** Yibo Gao, Dandan Cao, Mengfan Li, Fuqiang Zhao, Pei Wang, Shiwen Mei, Qianqian Song, Pei Wang, Yanli Nie, Wei Zhao, Sizhen Wang, Hai Yan, Xishan Wang, Yuchen Jiao, Qian Liu

**Affiliations:** 1 Central Laboratory & Shenzhen Key Laboratory of Epigenetics and Precision Medicine for Cancers, National Cancer Center/National Clinical Research Center for Cancer/Cancer Hospital & Shenzhen Hospital, Chinese Academy of Medical Sciences and Peking Union Medical College, Shenzhen, 518116 China; 2 Laboratory of Translational Medicine, National Cancer Center/National Clinical Research Center for Cancer/Cancer Hospital, Chinese Academy of Medical Sciences and Peking Union Medical College, Beijing, 100021 China; 3 Department of Thoracic Surgery, National Cancer Center/National Clinical Research Center for Cancer/Cancer Hospital, Chinese Academy of Medical Sciences and Peking Union Medical College, Beijing, 100021 China; 4 State Key Laboratory of Molecular Oncology, National Cancer Center/National Clinical Research Center for Cancer/Cancer Hospital, Chinese Academy of Medical Sciences and Peking Union Medical College, Beijing, 100021 China; 5 Institute of Cancer Research, Henan Academy of Innovations in Medical Science, Zhengzhou, 450000 China; 6 Department of Gastroenterology, Shanxi Province Cancer Hospital/Shanxi Hospital Affiliated to Cancers Hospital, Chinese Academy of Medical Sciences/Cancer Hospital Affiliated to Shanxi Medical University, Taiyuan, 030013 China; 7 Genetron Health (Beijing) Co. Ltd., Beijing, 102206 China; 8 Department of Colorectal Surgery, National Cancer Center/National Clinical Research Center for Cancer/Cancer Hospital, Chinese Academy of Medical Sciences and Peking Union Medical College, Beijing, 100021 China; 9 Department of Colorectal Surgery, Shanxi Province Cancer Hospital/Shanxi Hospital Affiliated to Cancers Hospital, Chinese Academy of Medical Sciences/Cancer Hospital Affiliated to Shanxi Medical University, Taiyuan, 030013 China

**Keywords:** Colorectal cancer, Early detection, Liquid biopsy, Multiomics

## Abstract

**Background:**

Early detection of colorectal cancer (CRC) significantly enhances patient outcomes. Conventional CRC screening tools, like endoscopy and stool-based tests, have constraints due to their invasiveness or suboptimal patient adherence. Recently, liquid biopsy employing plasma cell-free DNA (cfDNA) has emerged as a potential noninvasive screening technique for various malignancies.

**Methods:**

In this research, we harnessed the Mutation Capsule Plus (MCP) technology to profile an array of genomic characteristics from cfDNA procured from a single blood draw. This profiling encompassed DNA methylation, the 5’ end motif, copy number variation (CNV), and genetic mutations. An integrated model built upon selected multiomics biomarkers was trained using a cohort of 93 CRC patients and 96 healthy controls.

**Results:**

This model was subsequently validated in another cohort comprising 89 CRC patients and 95 healthy controls. Remarkably, the model achieved an area under the curve (AUC) of 0.981 (95% confidence interval (CI), 0.965–0.998) in the validation set, boasting a sensitivity of 92.1% (95% CI, 84.5%-96.8%) and a specificity of 94.7% (95% CI, 88.1%-98.3%). These numbers surpassed the performance of any single genomic feature. Importantly, the sensitivities reached 80% for stage I, 89.2% for stage II, and were 100% for stages III and IV.

**Conclusion:**

Our findings underscore the clinical potential of our multiomics liquid biopsy test, indicating its prospective role as a noninvasive method for early-stage CRC detection. This multiomics approach holds promise for further refinement and broader clinical application.

**Supplementary Information:**

The online version contains supplementary material available at 10.1186/s12943-024-01959-3.

## To the Editor,

Colorectal cancer (CRC) is one of the most common and lethal cancers worldwide [1]. While early detection can drastically improve patient outcomes [2], current colonoscopy methods pose limitations, such as invasiveness, patient discomfort, and resource constraints, making blood-based tests a preferable alternative for regular CRC screening [3, 4].

The emergence of liquid biopsies using cell-free DNA (cfDNA) has opened a promising avenue for managing cancers, including early-stage cancer detection. A pioneering test, Galleri, which uses cfDNA methylation to identify cancer signals, is commercially available for screening of more than 50 types of cancers in adults with an elevated risk. Although not FDA-approved yet, its significance has been demonstrated by large-scale real-world data [5, 6]. As for CRC, while many studies have focused on specific biomarkers like gene mutations, DNA methylation, or genome-wide features, few have attempted to compare or combine these different biomarkers for better accuracy in detection of caner [7–9]. This study leverages the Mutation Capsule Plus (MCP) technology to profile multiple genomic features simultaneously, aiming to develop a comprehensive multiomics assay for the early detection of CRC [10].

## Results

### Study design and characteristics of participants

**Fig. 1:**
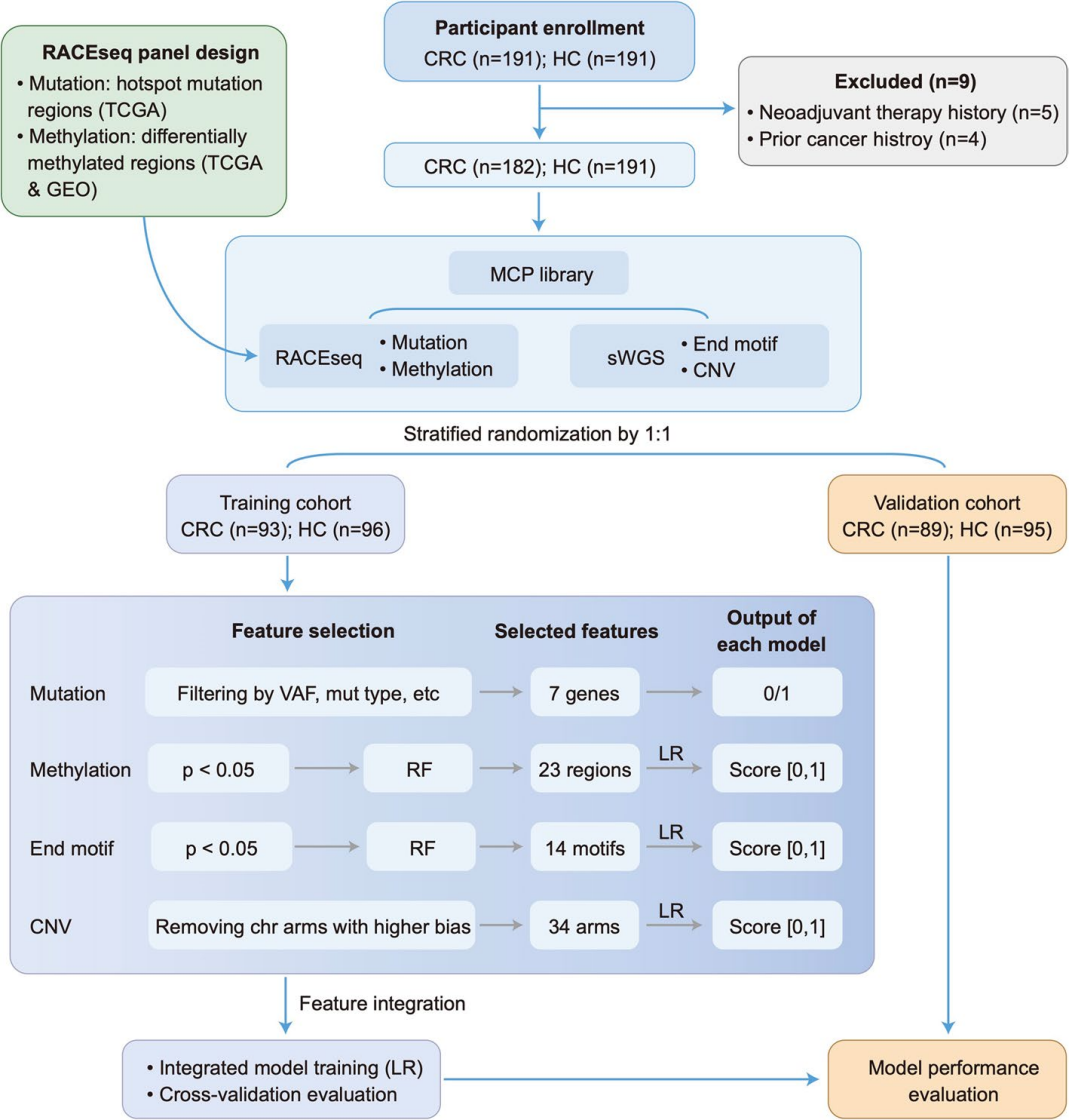
Workflow of the study. This study consists of three steps: panel design, model training and validation. In the panel design step, potential mutation and methylation markers were selected using public database. In the training cohort, feature selection and model construction were separately performed for each type of individual genomic feature. Then, an integrated model was constructed, which used the outputs of each individual model as inputs to generate the final prediction model. The integrated model was subsequently validated in the validation cohort. HC, healthy controls. RF, random forest. LR, logistic regression

The workflow of our molecular analysis and modeling for both the training (*n* = 189) and validation (*n* = 184) cohorts is depicted in Fig. 1. The age and sex ratio were balanced for CRC and healthy groups in the training cohort to reduce the impact of potential confounding factors. The training cohort included 93 CRC patients and 96 healthy individuals (Table S1). The proportion of stage I-III CRC patients was greater than the proportion of stage IV patients: 17.2%, 39.8%, and 38.7% for stage I-III patients, respectively, and 2.2% for stage IV patients. The validation cohort included 89 CRC patients and 95 healthy individuals and the clinical characteristics are shown in Table S1.

### Assay development and marker selection

Employing MCP technology, we managed to profile mutation, methylation and genome-wide features concurrently for each cfDNA sample [10]. First, a pre-MCP library was constructed. Briefly, cfDNA sample was digested with HhaI, a restriction endonuclease sensitive to CpG methylation, before ligation to customized adapters and whole genome amplification. The whole genome library (pre-MCP library) allows multiple downstream assays without sacrificing sensitivity like splitting limited cfDNA samples directly into several different reactions. Next, based on pre-MCP library, we performed shallow whole-genome sequencing (sWGS) to assess genome-wide signals such as arm-level copy number variation (CNV) and the 5’ end motif. We also profiled mutations and methylation changes in parallel from the pre-MCP library by targeted sequencing (RACEseq).

To determine the RACEseq panel for cfDNA profiling, we selected potential methylation markers based on the publicly available datasets from The Cancer Genome Atlas (TCGA) and the Gene Expression Omnibus (GEO). The sites that were hypermethylated in tumor tissues compared with white blood cells from healthy individuals and/or normal colorectal tissues from healthy and CRC individuals were selected for the panel (Methods). The panel for mutation profiling included the hotspot mutation regions in the driver genes frequently mutated in CRC, such as *APC*, *TP53*, *KRAS*, *PIK3CA*, *FBXW7*, and *BRAF*. We also included *ACVR2A*, a frequently mutated gene in hypermutated CRC, to cover diverse CRC subtypes (Table S2) [11].

**Fig. 2:**
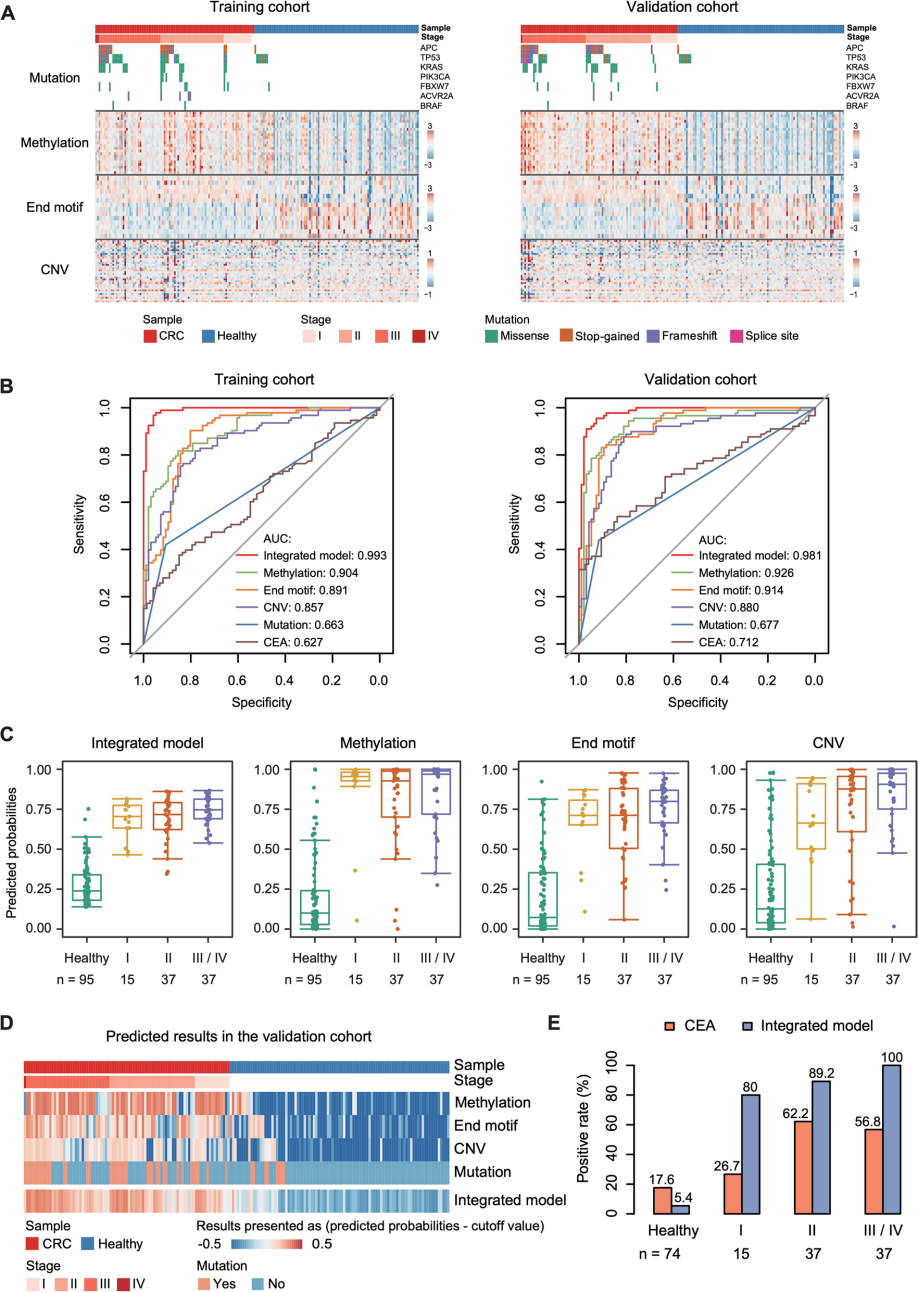
Marker profiles and model performance. **A** Feature profiles in the training and validation cohorts. The heatmaps display the selected biomarkers, including the mutation status of 7 genes, 23 DNA methylation markers, 14 5’ end motifs, and CNVs in 34 chromosome arms. The data of DNA methylation, end motif and CNV in the heatmaps were centered and scaled in the row direction. **B** Receiver operating characteristic (ROC) curves for the integrated model (integrating DNA methylation, 5’ end motif, CNV and gene mutation), each individual model (DNA methylation, 5’ end motif, CNV, or gene mutation) and plasma CEA assay in the training (left) and validation (right) cohorts. The plasma samples of 93 CRC and 73 healthy participants in the training cohort, and 89 CRC and 74 healthy participants in the validation cohort were available for the plasma CEA assay. **C** The predicted probabilities for healthy individuals and stage I-IV CRC patients predicted by the integrated model (integrating DNA methylation, 5’ end motif, CNV and gene mutation) and each individual model (DNA methylation, 5’ end motif or CNV) in the validation cohort. Participants with predicted probabilities close to 1 are more likely to have CRC based on the models. **D** The predicted results, presented as the probabilities predicted by each model minus the corresponding cutoff values (0.63 for DNA methylation model, 0.61 for end motif model, 0.81 for CNV model, and 0.51 for the integrated model), in the validation cohort. The reddish signals indicate the sample is predicted to be CRC positive based on the corresponding model, while the bluish signals indicate the sample is predicted to be normal/CRC negative based on the corresponding model. **E** The positive rates for plasma CEA and CA19-9 assays and our integrated CRC screening model in healthy individuals and stage I-IV CRC patients in the validation cohort. The plasma samples of 89 CRC and 74 healthy participants in the validation cohort were available for the plasma protein assay

Using the RACEseq and sWGS assays, we profiled the molecular features of the pre-MCP libraries in the training cohort and further selected the most informative biomarkers with the potential to distinguish CRC from healthy individuals. To reduce false positive mutations, we applied stringent filtering criteria, including mutation type, mutation hotspot, mutant frequency in the COSMIC database and the variant allele frequency (VAF) (Methods). The mutation test detected at least one eligible mutation in 41.9% (39/93) of CRC patients and 9.4% (9/96) of healthy participants in the training cohort (Fig. 2A, Table S3). Specifically, mutations in *APC*, *TP53* and *KRAS* accounted for a large proportion of mutation-positive CRC patients, detecting 19.4% (18/93), 23.7% (22/93) and 17.2% (16/93) of CRC patients, respectively. Mutations in *FBXW7* and *ACVR2A* also contributed in identifying CRC patients, each detected an additional 3.2% (3/93) of CRC patients, indicating the complementary role of these genes in the combination (Fig. 2A).

DNA methylation level of methylation target sites was calculated as the ratio of the number of methylated molecules versus the overall number of methylated and unmethylated molecules at corresponding position (Methods). The frequencies of each 5’ end motif were calculated as previously reported [12], and CNVs were measured by ichorCNA with recommended parameters for low tumor content samples [13]. DNA methylation, the 5’ end motif and CNV data were separately filtered, and the random forest algorithm was used for marker selection for DNA methylation and the 5’ end motif in the training cohort. After filtering and marker selection, 7 mutated genes, 23 DNA methylation markers, 14 end motifs, and CNVs in 34 chromosome arms demonstrated potential for distinguishing CRC patients from healthy individuals in the training cohort on the basis of cfDNA and were retained for subsequent modeling (Fig. 2A).

### Development and validation of the CRC multiomics screening model

Based on the selected markers, we constructed logistic regression models for each of the following data types, end motif, DNA methylation and CNV. With tenfold cross-validation in the training cohort, the probability scores predicted by each model exhibited potential for distinguishing CRC patients from healthy controls (Fig. 2B, S1). The area under the curve (AUC) ranged from 0.857 (95% confidence interval (CI), 0.804–0.910) for CNV, 0.891 (95% CI, 0.844–0.938) for end motif, to 0.904 (95% CI, 0.862–0.945) for DNA methylation. To enhance the differentiating power, we constructed a combined model by integrating the four genomic features. A mutation score of 1 or 0, which represents a sample with or without at least one eligible mutation, as well as probability scores predicted separately by the end motif, DNA methylation and CNV models, were input into a logistic regression model (Methods). With tenfold cross-validation, the integrated model achieved an AUC of 0.993 (95% CI, 0.985–1.000) in the training cohort, with a sensitivity of 97.8% (95% CI, 92.4%-99.7%) and a specificity of 94.8% (95% CI, 88.3%-98.3%) (Fig. 2B).

To verify the effectiveness of the integrated model, we evaluated its performance in a validation cohort of 184 participants, including 89 CRC patients and 95 healthy controls (Table S1). The integrated model exhibited a consistently strong screening potential, achieving an AUC of 0.981 (95% CI, 0.965–0.998), with 92.1% (95% CI, 84.5%-96.8%) sensitivity and 94.7% (95% CI, 88.1%-98.3%) specificity (Fig. 2B, C). The sensitivity improved with the CRC stage, which was 80% (12/15), 89.2% (33/37), 100% (36/36) and 100% (1/1) for stage I-IV CRC, respectively (Table S4). Among the individual features, DNA methylation yielded the best performance (AUC = 0.926, 95% CI 0.883–0.968), followed by end motif (AUC = 0.914, 95% CI 0.873–0.955) and CNV (AUC = 0.880, 95% CI 0.828–0.932) (Fig. 2B, C), similar to the results in the training cohort. We evaluated different feature combinations by training corresponding models and found that the integration of all 4 features was the optimal and most stable model (Fig. S2, Table S5). Different types of genomic features complement each other and contribute to the overall integrated model, suggesting the benefit of the multiomics screening method (Fig. 2D).

### Comparison of our multiomics model with conventional tumor biomarkers

In clinical practice, carcinoembryonic antigen (CEA) is commonly used tumor biomarker for CRC detection and prognosis. To demonstrate the clinical feasibility of our CRC early detection method, we assessed plasma CEA levels in the validation cohort and compared the performance of these assays to our integrated model. The sensitivity and specificity of CEA levels were 53.9% and 82.4%, respectively, and the sensitivities for stage I, II, III, and IV CRC patients were 26.7% (4/15), 62.2% (23/37), 55.6% (20/36), and 100% (1/1), respectively (Fig. 2E). These results suggest that our CRC early detection model outperformed conventional plasma CEA assay, demonstrating its potential for use in clinical practice (Fig. 2B, E).

## Discussion

Previous seminal studies have shown the superiority of methylation in multi-cancer early detection [14, 15]. In this study, we focused on a single cancer type, CRC, and found that integrating additional features substantially improved the overall performance (92.1% sensitivity and 94.7% specificity) compared with methylation alone (80.9% sensitivity and 91.6% specificity). Other features enhance methylation performance by providing complementary information (Fig. 2D). Moreover, this multi-omics test is based on targeted sequencing of selected loci and sWGS, which resulted in a limited cost increase compared with the single-omics assay, making it feasible for practical use.

The complementary and synergetic multiomics data in this study build on the MCP technology, which allows parallel profiling of multiple genomic features on a limited amount of plasma cfDNA, and thus improves the flexibility of the screening content. Each individual feature has variable power, with methylation having the highest, followed by end motif, CNV, and mutation. Among these features, methylation is particularly attractive in cancer early detection research, partly because it is a stable and pervasive epigenetic alteration that occurs frequently and early in tumorigenesis. Moreover, methylation patterns are less likely to be affected by genomic background noise, which may allow the detection of cancer-specific signals at lower tumor fraction levels. End motif is a novel fragmentomic feature that captures the global changes in the chromatin landscape of cancer cells, which are considered more stable and consistent than other local alterations. CNV performed worse in our study, possibly because it was derived from a very low depth of cfDNA sequencing data, happened less frequently, and was less specific (influenced by background noise and CHIP). The unsatisfactory performance of mutations is likely due to the rare tumor-derived mutant molecules circulating in the blood of early-stage cancer patients. In addition, mutations associated with CHIP in cancer-free individuals may also interfere with the performance. Sequencing of matched white blood cell DNA is an accurate but relatively costly way to rigorously remove the influence of CHIP on the detection of CNV and mutation [14].

To exclude the possibility of having other occult cancers or early-stage disease in our asymptomatic control samples, we were able to follow up 178/191 (93.2%) of participants in the control group and there was no cancer reported since the time of blood drawing. Despite the proof of concept of a multiomics CRC detection method, this study has several limitations. First, the limited sample size may affect the evaluation of model performance. Second, patients with colorectal precancerous lesions, which are also clinically significant concerns for CRC early detection programs, were not included in this study. Taken together, future prospective randomized studies with larger and more diverse cohorts are needed to further enhance and validate our model for CRC early detection.

In summary, we developed a blood-based method for early detection of CRC and demonstrated the screening potential of multiomics cfDNA-based biomarkers. With further validation, this multiomics strategy is expected to be implemented in clinical settings as a first-line screening modality prior to colonoscopy.

### Supplementary Information

**Supplementary Material 1.**

**Supplementary Material 2.**

## Data Availability

The data generated in this study are publicly available in Genome Sequence Archive for Human (GSA-Human, https://bigd.big.ac.cn/gsa-human/) at HRA002356. Reviewer access link: https://ngdc.cncb.ac.cn/gsa-human/s/Rr55SMw7.

## Abbreviations

CRC: Colorectal cancer
cfDNA: Cell-free DNA
MCP: Mutation Capsule Plus
CNV: Copy number variation
AUC: Area under the curve
CI: Confidence interval
sWGS: Shallow whole-genome sequencing
TCGA: The Cancer Genome Atlas
GEO: Gene Expression Omnibus
VAF: Variant allele frequency
CEA: Carcinoembryonic antigen
ROC: Receiver operating characteristic
HC: Healthy controls
RF: Random forest
LR: Logistic regression
