# Supplementary material for: Integration of multiomics features for blood-based early detection of colorectal cancer
Source: Mol Cancer. 2024 Aug 22;23:173. doi: 10.1186/s12943-024-01959-3 (PMC11340186; doi:10.1186/s12943-024-01959-3)
Supplement: Supplementary file 1 — Supplementary Material 1. [file 12943_2024_1959_MOESM1_ESM.docx]

**Supplementary Materials for**

**Integration of multiomics features for blood-based early detection of colorectal cancer**

Yibo Gao^1,2,3,4#*^, Dandan Cao^5#^, Mengfan Li^5#^, Fuqiang Zhao^6#^, Pei Wang^5^, Shiwen Mei^6^, Qianqian Song^4^, Pei Wang^4^, Yanli Nie^5^, Wei Zhao^6^, Sizhen Wang^5^, Hai Yan^5^, Xishan Wang^6*^, Yuchen Jiao^4*^, Qian Liu^6*^

^*^Correspondence should be addressed to Q.L. ([liuqian@cicams.ac.cn](mailto:liuqian@cicams.ac.cn)), Y.J. ([jiaoyuchen@126.com](mailto:jiaoyuchen@126.com)), X.W. ([wxshan1208@126.com](mailto:wxshan1208@126.com)) and Y.G. ([gaoyibo@cicams.ac.cn](mailto:gaoyibo@cicams.ac.cn)).

**This file includes:**

Supplementary Table S1, S4, S5

Supplementary Figure S1 to S2

Supplementary Materials and Methods

**Supplementary Tables**

**Table S1. Participant characteristics of the study**

|  | Training cohort (n=189) | | | Validation cohort (n=184) | | | |
| --- | --- | --- | --- | --- | --- | --- | --- |
|  | CRC  (n=93) | Healthy  (n=96) | *P* value | CRC  (n=89) | Healthy  (n=95) | *P* value |  |
| Age  (Mean ± sd) | 59.9 ± 11.2 | 58.3 ± 11.2 | 0.33 | 60.0 ± 10.9 | 58.9 ± 10.6 | 0.51 |  |
| Male / Female | 47 / 46  50.5% / 49.5% | 49 / 47  (51.0% / 49.0%) | 0.75 | 61 / 28  68.5% / 31.5% | 53 / 42  (55.8% / 44.2%) | 0.015 |  |
| Stage I | 16 (17.2%) |  |  | 15 (16.9%) |  |  |  |
| Stage II | 37 (39.8%) |  |  | 37 (41.6%) |  |  |  |
| Stage III | 36 (38.7%) |  |  | 36 (40.4%) |  |  |  |
| Stage IV | 2 (2.2%) |  |  | 1 (1.1%) |  |  |  |
| Stage NA | 2 (2.2%) |  |  |  |  |  |  |

*P* values for the differences between CRC and healthy groups (two-tailed t test for age, and two-tailed Chi-squared test for sex).

**Table S4. The predicted results of the integrated model in the validation cohort**

| Integrated model | Partici-pants | Predicted positive | Predicted negative | Sensitivity  (95% CI) | *P* value | Specificity  (95% CI) |
| --- | --- | --- | --- | --- | --- | --- |
| Healthy controls | 95 | 5 | 90 |  |  | 94.7%  (88.1%-98.3%) |
| CRC (All) | 89 | 82 | 7 | 92.1%  (84.5%-96.8%) |  |  |
| CRC (I-II) | 52 | 45 | 7 | 86.5%  (74.2%-94.4%) | < 0.001 |  |
| CRC (III-IV) | 37 | 37 | 0 | 100%  (90.5%-100%) |  |  |
| CRC (R) | 20 | 19 | 1 | 95.0%  (75.1%-99.9%) | < 0.001 |  |
| CRC (L) | 69 | 63 | 6 | 91.3%  (82.0%-96.7%) |  |  |
| CRC (MSI-H) | 7 | 7 | 0 | 100.0%  (59.0%-100%) | < 0.001 |  |
| CRC (MSS) | 82 | 75 | 7 | 91.5%  (83.2%-96.5%) |  |  |
| CRC (≤ 4 cm) | 46 | 41 | 5 | 89.1%  (76.4%-96.4%) | < 0.001 |  |
| CRC (> 4 cm) | 43 | 41 | 2 | 95.3%  (84.2%-99.4%) |  |  |

*P* values for the sensitivities in different CRC groups (two-tailed McNemar's Chi-squared test).

**Table S5. The model performance of different feature combinations in the validation cohort**

|  | AUC (95% CI) | Using fixed specificities | | Highest Youden’s index | |
| --- | --- | --- | --- | --- | --- |
|  |  | Sensitivity (95% CI) | Specificity (95% CI) | Sensitivity (95% CI) | Specificity (95% CI) |
| Integrated model | 0.981 (0.965–0.998) | 92.1% (84.5%-96.8%) | 94.7% (88.1%-98.3%) | 95.5% (88.9%-98.8%) | 92.6% (85.4%-97.0%) |
|  |  |  |  |  |  |
| Methylation | 0.926 (0.883-0.968) | 80.9% (71.2%-88.5%) | 91.6% (84.1%-96.3%) | 83.1% (73.7%-90.2%) | 90.5% (82.8%-95.6%) |
| End motif | 0.914 (0.873-0.955) | 78.7% (68.7%-86.6%) | 91.6% (84.1%-96.3%) | 84.3% (75.0%-91.1%) | 88.4% (80.2%-94.1%) |
| CNV | 0.880 (0.828-0.932) | 59.6% (48.6%-69.8%) | 91.6% (84.1%-96.3%) | 88.8% (80.3%-94.5%) | 80.0% (70.5%-87.5%) |
| Mutation | 0.677 (0.618-0.736) | 43.8% (33.3%-54.7%) | 91.6% (84.1%-96.3%) | 43.8% (33.3%-54.7%) | 91.6% (84.1%-96.3%) |
|  |  |  |  |  |  |
| Methylation + End motif | 0.973 (0.954-0.991) | 88.8% (80.3%-94.5%) | 94.7% (88.1%-98.3%) | 93.3% (85.9%-97.5%) | 91.6% (84.1%-96.3%) |
| Methylation + CNV | 0.965 (0.937-0.993) | 89.9% (81.7%-95.3%) | 94.7% (88.1%-98.3%) | 94.4% (87.4%-98.2%) | 92.6% (85.4%-97.0%) |
| Methylation + Mutation | 0.942 (0.907–0.978) | 76.4% (66.2%-84.8%) | 94.7% (88.1%-98.3%) | 86.5% (77.6%-92.8%) | 89.5% (81.5%-94.8%) |
| End motif + CNV | 0 .943 (0.909-0.976) | 82.0% (72.5%-89.4%) | 94.7% (88.1%-98.3%) | 91.0% (83.1%-96.0%) | 88.4% (80.2%-94.1%) |
| End motif + Mutation | 0.925 (0.887–0.963) | 53.9% (43.0%-64.6%) | 94.7% (88.1%-98.3%) | 84.3% (75.0%-91.1%) | 90.5% (82.8%-95.6%) |
| CNV + Mutation | 0.893 (0.845–0.941) | 42.7% (32.3%-53.6%) | 94.7% (88.1%-98.3%) | 89.9% (81.7%-95.3%) | 78.9% (69.4%-86.6%) |
|  |  |  |  |  |  |
| Methylation + End motif + CNV | 0.979 (0.961-0.997) | 92.1% (84.5%-96.8%) | 94.7% (88.1%-98.3%) | 94.4% (87.4%-98.2%) | 92.6% (85.4%-97.0%) |
| Methylation + End motif + Mutation | 0.975 (0.958–0.993) | 89.9% (81.7%-95.3%) | 94.7% (88.1%-98.3%) | 93.3% (85.9%-97.5%) | 93.7% (86.8%-97.6%) |
| Methylation + CNV + Mutation | 0.972 (0.950–0.995) | 89.9% (81.7%-95.3%) | 94.7% (88.1%-98.3%) | 89.9% (81.7%-95.3%) | 95.8% (89.6%-98.8%) |
| End motif + CNV + Mutation | 0.951 (0.921–0.980) | 83.1% (73.7%-90.2%) | 94.7% (88.1%-98.3%) | 92.1% (84.5%-96.8%) | 87.4% (79.0%-93.3%) |

**Supplementary Figures**


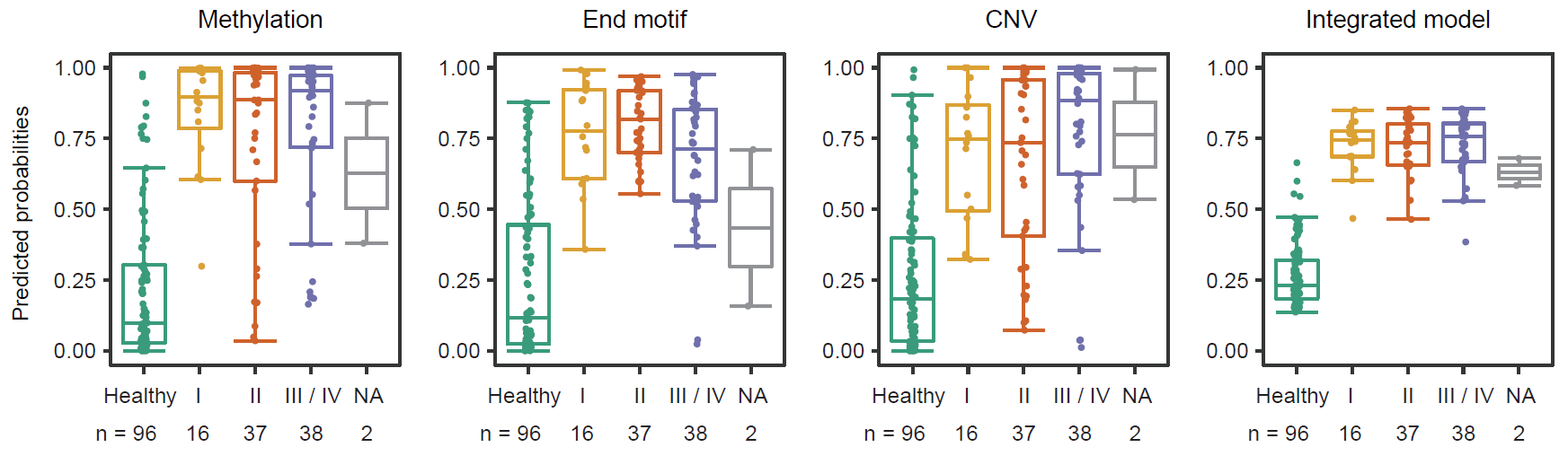
**Figure S1. The performance of each individual model and the integrated model in the training cohort.** The predicted probabilities for healthy individuals and stage I-IV CRC patients predicted by each individual model (DNA methylation, 5’ end motif or CNV) and the integrated model (integrating DNA methylation, 5’ end motif, CNV and gene mutation) in the training cohort. Participants with predicted probabilities close to 1 are more likely to have CRC based on the models.


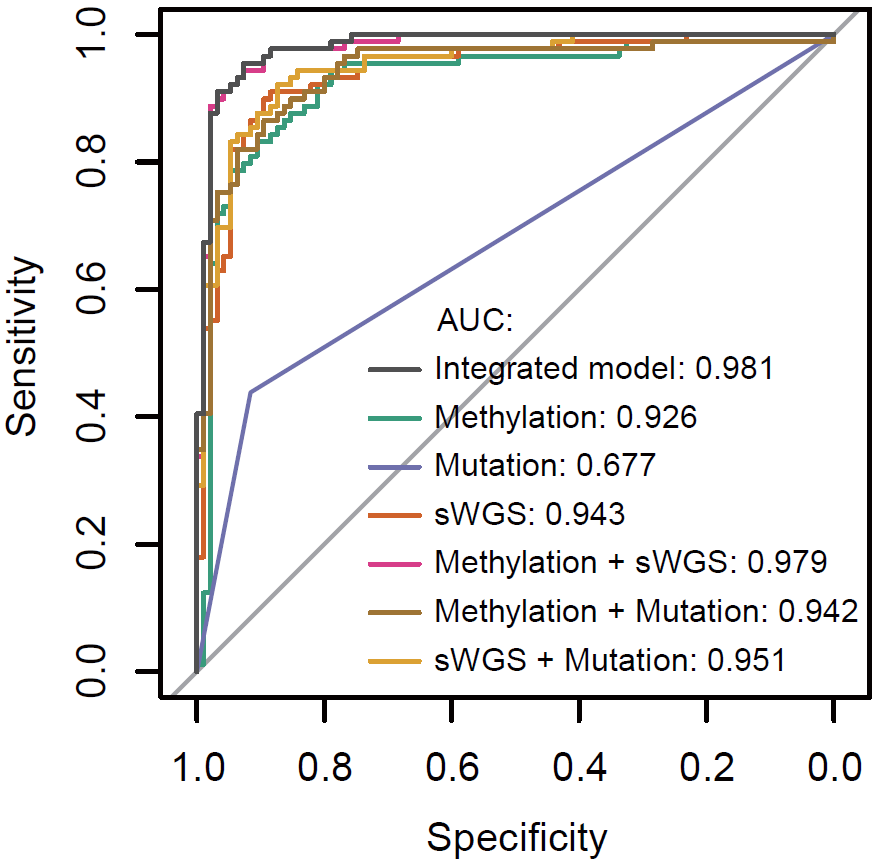


**Figure S2. ROC curves for models with different feature combinations in the validation cohort.** sWGS represents the combination of 5’ end motif and CNV.

**Supplementary Materials and Methods**

**Retrospective participant enrollment**

This study retrospectively collected and analyzed data from two groups of participants, colorectal cancer (CRC) patients and healthy controls. CRC patients were diagnosed and confirmed by histopathological examination following the current clinical practice guidelines in China; they were consecutively recruited from Cancer Hospital, Chinese Academy of Medical Sciences in Beijing, China, from September 2020 to November 2020. All patients were newly diagnosed and had not received any form of therapy before blood collection. Blood was withdrawn from patients before surgical removal of the tumor. The CRC pathological stage in Table S1 was determined according to the 8th American Joint Committee on Cancer (AJCC) TNM system after surgery. Healthy participants were matched with the CRC patients by age. None of the healthy participants had a diagnosis or past history of cancer at the time of blood collection. All participants were older than 18 years. Prior power analysis and blinding were not performed in this study.

**Sample collection and preprocessing**

For each participant, peripheral blood (10 mL) was collected with a cell-free DNA (cfDNA) collection tube from Streck (Streck; La Vista, NE, USA) and centrifuged at 2000 g for 15 min at 4°C to separate plasma and blood cells. Plasma was stored at -80°C until use. Before cfDNA extraction, plasma samples were further centrifuged at 16000 g for 10 min at 4°C to remove the residual cell debris, and cfDNA was extracted using a MiniMax High Efficiency cfDNA Isolation Kit (Apostle; San Jose, CA, USA) following the manufacturer’s instructions. The concentration of cfDNA was determined with the Qubit dsDNA HS Assay Kit (Thermo Fisher Scientific), and the Agilent 2100 Bioanalyzer system (Agilent; Santa Clara, CA, USA) was used to evaluate the DNA quality. Samples with hemolysis, coagulation, contamination of genomic DNA and insufficient amounts of cfDNA were excluded.

**Construction of the MCP library**

To detect multiomics data from a limited amount of cfDNA, we applied the Mutation Capsule Plus (MCP) technology [1]. First, the whole genome library (pre-MCP library) was constructed. Briefly, the methylation-sensitive restriction enzyme HhaI (5‘GCG^C3’, New England BioLabs, Ipswich, MA, USA) was used to selectively cut cfDNA. After end repair and A-tailing, customized MCP adapters containing unique molecular identifiers (UMIs) were ligated to DNA fragments. The fragments were amplified for 10 cycles. After purification and quantification, the pre-MCP library was used to conduct whole-genome sequencing (WGS) and RACEseq assays.

**WGS assay**

The pre-MCP library was amplified for 3 cycles, and Illumina sequencing adapters were added in this process. After purification and quantification, the library was sequenced on the Illumina NovaSeq 6000 system (Illumina; San Diego, CA, USA). Each sample was sequenced to a depth of approximately 2X.

**RACEseq assay**

We designed a RACEseq assay to profile mutations and methylation alterations in target genomic regions. For mutations, we targeted frequently mutated regions of frequently mutated genes based on the The Cancer Genome Atlas (TCGA) CRC dataset. These genes included *APC*, *TP53*, *KRAS*, *PIK3CA*, *FBXW7*, *ACVR2A* and *BRAF* [2,3]. As *APC* mutations are common early events in the development of CRC, and are not confined to hotspot regions but spread across the gene, we targeted approximately 3 kb regions of the *APC* gene in the RACEseq assay [2,4,5]. In addition, amplicons of the *TP53* gene in the assay covered genomic regions of about 1.3 kb. For the other 5 genes, only hotspot mutations were targeted, for example, G12, G13, Q61, A146 for *KRAS* and V600 for *BRAF* [2,6]. Overall, the RACEseq assay was designed to detect mutations in genomic regions covering 4,554 bp.

For methylation, we integrated Illumina Infinium Human Methylation 450K BeadChip data for colorectal tumor tissue, normal tissue adjacent to the tumor, colon mucosae from healthy donors, and white blood cell DNA from healthy individuals obtained from TCGA dataset, GSE40279 and GSE131013 datasets in the Gene Expression Omnibus (GEO) to enrich differentially methylated regions (DMRs) in CRC. The DMRs that overlapped with or near GCGC sites (HhaI recognition site) were integrated into the RACEseq panel.

The pre-MCP library was amplified through 3 cycles of PCR, and the amplified pre-MCP library served as the template for the RACEseq assay. The RACEseq assay involved two more rounds of PCR to efficiently amplify the target regions. In the first round of amplification, 9 cycles of PCR were performed on the amplified pre-MCP library with one universal primer matching the MCP adapters and one specific primer matching the target regions. A second round of PCR was performed for 14 cycles using a pair of nested primers matching the MCP adapters and the target regions to further enrich the library and to add Illumina sequencing adapters. After purification and quantification, the library was sequenced on the Illumina NovaSeq 6000 system (Illumina). Each sample was sequenced to approximately 10 G base pairs.

**CEA assay**

Plasma carcinoembryonic antigen (CEA) levels were determined using the ARCHITECT i1000SR immunoassay analyzer (Abbott Laboratories; Abbott Park, IL, USA) according to the manufacturer’s standard protocols. Two replicates were performed for each sample. The average value of the two replicates was used to determine the protein status. Plasma CEA less than 3.3 ng/mL was identified as normal.

**WGS data preprocessing**

Adapters and low-quality bases were first trimmed with Trimmomatic software [7]. Clean reads were aligned to the human reference genome hg19 using BWA [8] with default parameters. Reads with mapping quality < 20 were removed. Reads were then collapsed into original unique cfDNA molecules (read families) based on their coordinates, fragment size and UMI. The deduplicated bam file, which contains reads that represent unique read families, was downsampled to 13 M read pairs (~1.3X) and then used in the 5’ end motif quantification and copy number variation (CNV) calling steps.

End motifs were identified using the first 4-mer sequence on each 5’ end of paired-end reads. After removing all fragments overlapping with the Hhal restriction site GCGC, we calculated the frequency of each 4-mer motif.

CNVs were determined with ichorCNA [9] following the parameters recommended for low tumor fraction plasma samples. To mitigate technical bias (*e.g.* sequencing variations), 154 independent healthy samples were used to build the reference control set. For each sample, we divided the genome into 1-Mb nonoverlapping bins and calculated the log2 ratios relative to the reference control set. Bin-level log2 ratios were further aggregated to represent chromosome arm signals.

**RACEseq data preprocessing**

For methylation, the steps for adapter trimming, alignment, read filtering and collapsing into unique read families were the same as for WGS. Read families with at least two supporting reads were retained as effective read families. For each targeted GCGC site, the methylation levels were defined as the number of effective methylated read families (fully covering the HhaI cutting site) divided by the sum of the number of effective methylated read families and the number of effective unmethylated read families (ended at the HhaI cutting site).

For mutation, the steps of adapter trimming, alignment, and read filtering were the same as for WGS. SAMtools mpileup [10] was used for mutation calling across the targeted regions. Reads were collapsed into read families based on their coordinates, fragment size and UMI. Read families with at least two supporting reads and in which > 80% of reads were the same type (supporting a mutation or not) were retained as effective read families and used for the calculation of variant allele frequency (VAF). The effective read families supporting mutations were further manually reviewed in Integrative Genomics Viewer (IGV) [11].

**Marker selection**

For DNA methylation and the 5’ end motif, the Wilcoxon rank-sum test was used to compare the CRC and healthy groups in the training cohort. Markers with p values lower than 0.05 (two-sided) were considered to be significantly different between the two groups. The random forest method was applied to further reduce the number of biomarkers. For CNVs, chromosome arms with higher bias of depth, such as 19p and 19q, were excluded. Eventually, 23 DNA methylation regions, 14 5’ end motifs (4-mer) and CNVs in 34 chromosome arms were retained for model construction.

The detected mutations were filtered considering mutation type, mutation hotspot, mutant frequency in the COSMIC database and the VAF [6]. First, only mutations with distinct CRC signatures were retained: 1) For *APC*, only truncating mutations were retained, because more than 90% of *APC* mutations in CRC patients are the truncating type, while *APC* mutations associated with clonal hematopoiesis of indeterminate potential (CHIP) are mainly the missense type [2,12,13]. 2) For *TP53*, we divided the detected mutations into high-confidence hotspot mutations and other mutations, and applied different cutoffs in the VAF filtering step (see below), considering that CRC hotspot mutations were more likely to originate from tumors than CHIP. High-confidence hotspot mutations were defined as those with a mutant frequency greater than 1% in either the COSMIC targeted screen data or the COSMIC genome screen data of CRC [6]. 3) For *BRAF*, only V600E mutations were retained. 4) For *KRAS*, *PIK3CA*, *FBXW7* and *ACVR2A*, detected mutations in the mutation hotspot regions included in this assay were all retained. For these candidate mutations, a VAF filtering step was then applied, in which the customized VAF cutoff values were determined based on the training cohort. The customized VAF cutoff values were the following: 0.002 for *APC* truncating mutations, 0.0015 for *TP53* high-confidence hotspot mutations, 0.003 for other mutations in *TP53*, and 0.001 for mutations in the other 5 genes.

**Model construction**

For DNA methylation, arm-level CNVs and 5’ end motifs, logistic regression model was performed for individual genomic feature-based model construction in the training cohort. The parameters of the models were optimized through 10-fold cross-validation within the training cohort. The probability score of each genomic feature for each sample, which ranged from 0 to 1, was generated by the individual genomic feature-based models, in which a higher score represented a higher probability for cancer. The probability scores of DNA methylation, arm-level CNV, and 5’ end motif models, and mutation scores of 1 or 0, representing samples with or without eligible mutations, were used to create the final multidimensional model through another logistic regression model and produce a comprehensive probability score. Cutoff values and the sensitivities of models in the validation cohort were determined by the highest Youden’s index score, or at a specific specificity (91.6% for individual genomic feature-based models and 94.7% for feature combination models) to facilitate the comparison of the different models. A 10-fold cross-validation method was used to evaluate the model performance in the training cohort.

**Plots and statistical analysis**

The R software environment [14] was used for statistical computing and graphics.

**References**

1. Wang P, Song Q, Ren J, Zhang W, Wang Y, Zhou L, et al. Simultaneous analysis of mutations and methylations in circulating cell-free DNA for hepatocellular carcinoma detection. Sci Transl Med. 2022;14:eabp8704.

2. Martínez-Jiménez F, Muiños F, Sentís I, Deu-Pons J, Reyes-Salazar I, Arnedo-Pac C, et al. A compendium of mutational cancer driver genes. Nat Rev Cancer. 2020;20:555–72.

3. The Cancer Genome Atlas. Comprehensive molecular characterization of human colon and rectal cancer. Nature. 2012;487:330–7.

4. Fearon ER, Vogelstein B. A genetic model for colorectal tumorigenesis. Cell. 1990;61:759–67.

5. Aitchison A, Hakkaart C, Day RC, Morrin HR, Frizelle FA, Keenan JI. APC Mutations Are Not Confined to Hotspot Regions in Early-Onset Colorectal Cancer. Cancers (Basel). 2020;12.

6. Tate JG, Bamford S, Jubb HC, Sondka Z, Beare DM, Bindal N, et al. COSMIC: the Catalogue Of Somatic Mutations In Cancer. Nucleic Acids Res. 2019;47:D941–7.

7. Bolger AM, Lohse M, Usadel B. Trimmomatic: a flexible trimmer for Illumina sequence data. Bioinformatics. 2014;30:2114–20.

8. Li H. Aligning sequence reads, clone sequences and assembly contigs with BWA-MEM. 2013 [cited 2021 Dec 13]; Available from: https://arxiv.org/abs/1303.3997v2

9. Adalsteinsson VA, Ha G, Freeman SS, Choudhury AD, Stover DG, Parsons HA, et al. Scalable whole-exome sequencing of cell-free DNA reveals high concordance with metastatic tumors. Nat Commun. 2017;8:1324.

10. Li H, Handsaker B, Wysoker A, Fennell T, Ruan J, Homer N, et al. The Sequence Alignment/Map format and SAMtools. Bioinformatics. 2009;25:2078–9.

11. Thorvaldsdóttir H, Robinson JT, Mesirov JP. Integrative Genomics Viewer (IGV): high-performance genomics data visualization and exploration. Brief Bioinform. 2013;14:178–92.

12. Rowan AJ, Lamlum H, Ilyas M, Wheeler J, Straub J, Papadopoulou A, et al. APC mutations in sporadic colorectal tumors: A mutational “hotspot” and interdependence of the “two hits”. Proc Natl Acad Sci U S A. 2000;97:3352–7.

13. Liu J, Chen X, Wang J, Zhou S, Wang CL, Ye MZ, et al. Biological background of the genomic variations of cf-DNA in healthy individuals. Ann Oncol Off J Eur Soc Med Oncol. 2019;30:464–70.

14. R Core Team. R: A Language and Environment for Statistical Computing [Internet]. Vienna, Austria: R Foundation for Statistical Computing; 2021. Available from: http://www.r-project.org/
